# The sequence capture by hybridization: a new approach for revealing the potential of mono‐aromatic hydrocarbons bioattenuation in a deep oligotrophic aquifer

**DOI:** 10.1111/1751-7915.12426

**Published:** 2016-10-21

**Authors:** Magali Ranchou‐Peyruse, Cyrielle Gasc, Marion Guignard, Thomas Aüllo, David Dequidt, Pierre Peyret, Anthony Ranchou‐Peyruse

**Affiliations:** ^1^ Université de Pau et des Pays de l'AdourEquipe Environnement et Microbiologie, IPREM‐CNRS 5254F‐64013PauFrance; ^2^ Université d'AuvergneEA 4678 CIDAM63001Clermont‐FerrandFrance; ^3^ TIGF – Transport et Infrastructures Gaz France40 Avenue de l'Europe, CS2052264000PauFrance; ^4^ STORENGY – Geosciences DepartmentBois‐ColombesFrance

## Abstract

The formation water of a deep aquifer (853 m of depth) used for geological storage of natural gas was sampled to assess the mono‐aromatic hydrocarbons attenuation potential of the indigenous microbiota. The study of bacterial diversity suggests that Firmicutes and, in particular, sulphate‐reducing bacteria (*Peptococcaceae*) predominate in this microbial community. The capacity of the microbial community to biodegrade toluene and *m*‐ and *p*‐xylenes was demonstrated using a culture‐based approach after several hundred days of incubation. In order to reveal the potential for biodegradation of these compounds within a shorter time frame, an innovative approach named the solution hybrid selection method, which combines sequence capture by hybridization and next‐generation sequencing, was applied to the same original water sample. The *bssA* and *bssA*‐like genes were investigated as they are considered good biomarkers for the potential of toluene and xylene biodegradation. Unlike a PCR approach which failed to detect these genes directly from formation water, this innovative strategy demonstrated the presence of the *bssA* and *bssA*‐like genes in this oligotrophic ecosystem, probably harboured by *Peptococcaceae*. The sequence capture by hybridization shows significant potential to reveal the presence of genes of functional interest which have low‐level representation in the biosphere.

## Introduction

The degradation of toluene and *m*,* p* and *o*‐xylenes (TX) under anoxic conditions has been demonstrated in numerous marine and continental environments. It can be associated with the reduction of nitrate, sulphate, iron and CO_2_ (Dolfing *et al*., 1990; Beller and Spormann, 1997; Harms *et al*., 1999; Kane *et al*., 2002; Kube *et al*., 2004; Morasch *et al*., 2004; Morasch and Meckenstock, 2005; Washer and Edwards, 2007; Aüllo *et al*., 2016). In all cases, the initial addition of fumarate to the hydrocarbon molecule is catalysed by either benzylsuccinate synthase (toluene) or benzylsuccinate synthase‐like enzymes (xylenes). For about 15 years, the *bssA* gene, which encodes for the alpha subunit of this protein, has been used as a biomarker for the biodegradation of TX under anoxic conditions. Beller *et al*. (2002) were the first to design primers targeting this gene, using available sequences from isolated strains. These primers then preferentially targeted nitrate‐reducing Betaproteobacteria. Subsequently, other primer sets enabled sulphate‐reducing, iron‐reducing and syntrophic bacteria to be targeted (Winderl *et al*., 2007; Beller *et al*., 2008; Staats *et al*., 2011; Fowler *et al*., 2014). There was a difficulty in amplifying the *bssA* gene of some sulphate‐reducing bacteria belonging to the *Clostridia* which was partly solved by the subsequent design of specific primers for this class (von Netzer *et al*., 2013; for the latest review on *bssA*‐like gene diversity, see von Netzer *et al*., 2016).

In oligotrophic and stable environments, like deep continental aquifers (−500 to −1200 m), the input of exogenous TX represents a potential source of carbon which is likely to modify the growth and survival strategies of microorganisms. Over the last several years, the potential for biodegradation of monoaromatic hydrocarbons in such environments has been demonstrated through culture‐based approaches (Morasch *et al*., 2004; Berlendis *et al*., 2010; Aüllo *et al*., 2016). The low biomass present in these ecosystems makes the demonstration of biodegradation capacities difficult (i.e. detection of *bssA* genes) in formation water (FW). It is currently difficult, indeed impossible, to assess the degradation potential of FW sampled from a deep aquifer without having recourse to laboratory‐based degradation assays. In our previous studies, amplifications of the *bssA* gene using the primers cited previously have unfortunately often proved to be unsuccessful, as a result of the few targets available and problems of non‐specific amplification (Aüllo *et al*., 2016). Next‐generation sequencing (NGS) techniques allow a deeper analysis of genetic diversity and thus offer the possibility of dispensing with culture‐based techniques. However, diversity analyses are very often unable to identify microbial populations with low‐level representation. The sequence capture by hybridization (Gasc *et al*., 2016), therefore, constitutes an alternative for the efficient detection of rare or unknown sequences in metagenomic samples (Denonfoux *et al*., 2013; Bragalini *et al*., 2014; Biderre‐Petit *et al*., 2016).

In the context of this study, we sought to demonstrate the presence of *bssA* genes in a deep aquifer used to store natural gas, which is associated with trace amounts of other hydrocarbons, in order to reveal a potential for natural bioattenuation of TX. We hypothesized that the *Clostridia*, in particular members of the *Peptococcaceae* family, play a key role in the degradation of TX in deep aquifers (Basso *et al*., 2009; Berlendis *et al*., 2010; Aüllo *et al*., 2016). It is known that some members of *Peptococcaceae* are capable of degrading mono‐aromatic hydrocarbons directly or via syntrophic relationships (Morasch *et al*., 2004; Taubert *et al*., 2012). A conventional approach to amplify the *bssA* gene using the sets of primers available in the literature is compared with the sequence capture by hybridization approach. This study represents the first instance of its use in this type of environment and in an industrial context.

## Materials and methods

### Sampling

In 2011, water samples were obtained from a deep aquifer (853 m of depth) used for geological storage of natural gas (aquifer 1, Paris Basin, France). Several physicochemical parameters of the FW are indicated in Table 1 (IPL Santé, Environnement Durables, Ile De France). After the tubing was cleaned as described previously (Basso *et al*., 2005), the biomass from the FW was collected at the wellhead by filtration through 70 Sterivex^®^ filters (EMD; Millipore, Molsheim, France), while maintaining anoxic conditions. Filters were used to collect the microbial biomass over a period of 6.5 h from 500 l of FW. At the end of sampling, the filters were placed in bags maintaining anaerobiosis (GasPak^™^ EZ; BD, Franklin Lakes, NJ, USA). Several litres of FW were also sampled in sterile glass bottles and degassed with nitrogen in the laboratory prior to subsequently preparing and running microbial cultures. The set of samples (filters and water) were immediately placed at 4°C and transported to the laboratory. The samples to be used for molecular analysis were then frozen at −20°C and those for the cultures used the following day.

**Table 1:** Physico‐chemical parameters and constituents of the formation water sampled from a deep aquifer (aquifer 1 in this study) at 853 m of depth below groundwater and analysed at atmospheric pressure

Physico‐chemical parameters	
Temperature (°C)	36
pH	8.25
Conductivity at 25°C (μS cm^−1^)	6000
Redox potential (mV)	−363
Pressure (bars)	93
Total suspended solids (mg l^−1^)	16.0
Constituents^a^	
Carbonates (mg l^−1^)	< 20
Sulphates (mg l^−1^)	2186.5
Ammonium (mg l^−1^)	1.75
Calcium (mg l^−1^)	25.1
Magnesium (mg l^−1^)	15.9
Sodium (mg l^−1^)	1400
Potassium (mg l^−1^)	34.0
Chloride (mg l^−1^)	260
Silicates (mg SiO_2_ l^−1^)	15.8
Phosphorus (mg l^−1^)	< 0.05
Nitrates (mg l^−1^)	< 2
Fluoride (mg l^−1^)	1.51
Barium (mg l^−1^)	0.013
Total iron (μg l^−1^)	3200
Ferrous iron (μg l^−1^)	< 100
Manganese (μg l^−1^)	44
Organic carbon (mg l^−1^)	1.0

a Arsenic, cadmium, chrome, copper, tin, mercury, lead, vanadium and zinc were also measured, but were below the limits of detection.

### Biodegradation assays

Two biodegradation assays of a mixture of toluene, *m*‐, *p*‐ and *o*‐ xylenes, here referred to as TX (with a final concentration of 10 ppm for each hydrocarbon; Sigma‐Aldrich, Saint Quentin Fallavier, France) were carried out at the same time with FW alone (FW condition) or enriched with the concentrated biomass (FWCB condition). The concentrated biomass was obtained for the latter condition by resuspending 67 Sterivex^®^ filters (EMD; Millipore) in 250 ml of anoxic FW under agitation. Ten per cent (v/v) of this concentrated biomass was used to inoculate the FWCB. For each condition, that is, without addition of concentrated biomass (FW+TX) and with addition of concentrated biomass (FWCB+TX), an abiotic control was performed by the addition of 5% v/v of 1 M HCl. The four microcosms, with a final volume of 40 ml, were prepared in 100 ml Wheaton serum bottles sealed with butyl rubber stoppers (Bellco Glass, Vineland, NJ). All manipulations were performed in a glovebox (Getinge La Calhene, France) in an atmosphere of 95% N_2_ and 5% H_2_. The hydrogen is necessary for the palladium catalyst can react to remove oxygen traces. All cultures were incubated at 37°C, in the dark without agitation. Periodically, TX degradation was monitored in the four microcosms using SPME/GC/FID as described in Aüllo *et al*. (2016).

### Bacterial enumeration

Eighteen millilitres of FW were fixed on site with 2 ml 10% borax‐buffered formaldehyde (37%; Sigma‐Aldrich) and stored at 4°C until quantification. Furthermore, after resuspension of the Sterivex^™^ filters in FW for the biodegradation assays, 18 ml of the concentrated biomass was sampled and fixed the same way. In all cases, 500 μl of 4′,6′‐diamidino‐2‐phenylindole (DAPI; Sigma‐Aldrich) stock solution (200 μg ml^−1^) was added to 10 ml of fixed sample, and then vacuum filtration was performed using 0.2 μm pore‐size black polycarbonate filters (Millipore). Ten fields were selected at random for each filter, and the cells were counted on an Axio Observer.Z1 inverted microscope (Zeiss, Oberkochen, Germany) equipped with a 63× oil immersion objective (Plan APO, N.A. 1.4, M27). Images were obtained with a Zeiss Axiocam 506 mono CCD camera via the Zeiss ZEN 2012 interface.

### Bacterial community analysis

Microbial diversity was investigated on a Sterivex^™^ filter previously stored at −20°C. The filter was crushed in liquid nitrogen and the DNA extracted with the Powersoil DNA Isolation kit (MoBio Laboratories, Inc., Carlsbad, CA, USA) following the manufacturers' specifications. Genomic DNA was sent to a commercial company (MR DNA, Shallowater, TX, USA). The hypervariable V4 region of the 16S rRNA gene was amplified with PCR primers 515/806 and sequenced by the MiSeq 2 × 300 bp run (Illumina, San Diego, CA, USA). The construction of DNA libraries with the amplicons and the sequencing were performed following the manufacturers' specifications. The sequence data were subsequently processed through the analysis pipeline designed by MR DNA. At first, the sequences were demultiplexed and the barcodes and primers were removed before eliminating all sequences smaller than 150 bp. Sequences displaying ambiguous bases were also eliminated. Operational taxonomic units (OTUs) were defined by clustering sequences displaying 97% similarity. Thus, singleton sequences and chimeras were eliminated. The final OTUs were subsequently classified taxonomically using BLASTn against a curated database derived from RDPII and NCBI.

### Cloning and sequencing of benzylsuccinate synthase alpha‐subunit (*bssA*) genes

PCR amplifications of the *bssA* gene were performed for different sets of primers found in the literature. The primer sets 7772F/8546R (Winderl *et al*., 2007), 7768F/8543R (von Netzer *et al*., 2013), bss3F/*bssA*R (Staats *et al*., 2011), and 997F/1230R (Brow *et al*., 2013) were used to target, respectively, the *bssA* gene of Deltaproteobacteria, Clostridia, *Geobacter* and nitrate‐reducing Betaproteobacteria (Table S1). For each positive amplification, the PCR fragment corresponding to the expected size was purified on an agarose gel and cloned using the TOPO TA Cloning kit (Fisher Scientific, Hampton, NH, USA) before being sequenced by Qiagen Genomic Services (Hilden, Germany).

### Capture probe design and synthesis

A set of five 31‐ to 38‐mers degenerate probes covering the *bssA* gene was designed from nine *bssA* nucleic sequences from strains belonging to Deltaproteobacteria, Firmicutes (EF123665, EF123667, EU780921, FO203503, EF123663, EF123662) and environmental sequences obtained with 7772F/8546R primers set. These three latter amplicons were obtained at the end of TX degradation from enrichment cultures with autochthonous microbiota of different deep aquifers (aquifer 1: KX576576, aquifer 2: KX576577, aquifer 3: KX576575). The deep aquifer 2 was studied by Berlendis *et al*. (2010) (Paris Basin). The third aquifer (aquifer 3) is located in the Aquitaine Basin (southwest of France). These sequences were processed using the KASpOD software (Parisot *et al*., 2012) (Table S1). Adaptor sequences were added at each extremity of the probe to enable their PCR amplification, resulting in “ATCGCACCAGCGTGT‐N_(31‐38)_‐CACTGCGGCTCCTCA” sequences, with N_(31‐38)_ representing the *bssA*‐specific capture probe. Biotinylated RNA capture probes were then synthesized as described by Ribière *et al*. (2016).

### Preparation of biological samples and libraries

Next‐generation sequencing libraries were constructed on genomic DNA extracted directly from aquifer 1 FW after concentration by means of ethanol precipitation. Libraries were prepared using the Nextera XT Kit (Illumina) using the manufacturer's instructions (Genoscreen, Lille, France) and were PCR amplified using the GC‐RICH PCR system kit (RocheDiagnostics GmbH, Mannheim, Germany) with primers complementary to the library adapters to obtain sufficient amounts of DNA to run the sequence capture by hybridization.

### Hybridization capture and elution

Solution hybrid selection (SHS) was conducted on FW sample according to the protocol described by Ribière *et al*. (2016). Briefly, 500 ng of heat denatured libraries was hybridized to the set of biotinylated RNA probes for 24 h at 65°C. Probe/target heterodimers were trapped by streptavidin‐coated paramagnetic beads (Dynabeads M‐280 Steptavidin, Invitrogen, Carlsbag, CA, USA). After several washing steps, the captured targets were eluted from the beads using NaOH and purified using AMPure beads (Beckman Coulter Genomics, Takeley, Essex, United Kingdom). Enriched products were PCR amplified using primers complementary to the library adapters and purified again using AMPure beads (Beckman Coulter Genomics). To increase the enrichment, a second round of hybridization and amplification was performed using the obtained captured products.

### Illumina MiSeq sequencing and data analysis

Captured DNA products were sequenced using a single MiSeq 2 × 300 bp run (Illumina) according to the manufacturer's specifications (Genoscreen). All raw reads were scanned for library adaptors and quality filtered using PRINSEQ‐lite PERL script (Schmieder and Edwards, 2011) prior to assembly and analysis. The clean reads were assembled de novo using IDBA‐UD (v1.1.1) (Peng *et al*., 2012). Contigs generated were combined for a second round of assembly using CAP3 to generate longer contigs (Huang and Madan, 1999). The amino acid (AA) sequences were deduced from the final assembled nucleotide contigs and then aligned with reference open‐reading frames sourced from public databases using MEGA version 6 (Tamura *et al*., 2013). The phylogenetic tree was constructed with the same software using the neighbour‐joining method. The bootstrap analysis was performed for 1000 replicates.

### Nucleotide sequence accession numbers

Sixty‐five sequences (*16S rRNA* and *bssA genes*) have been submitted to GenBank under accession numbers KX576572 to KX576636.

## Results and discussion

### Characterization of the site

**Figure 1:**
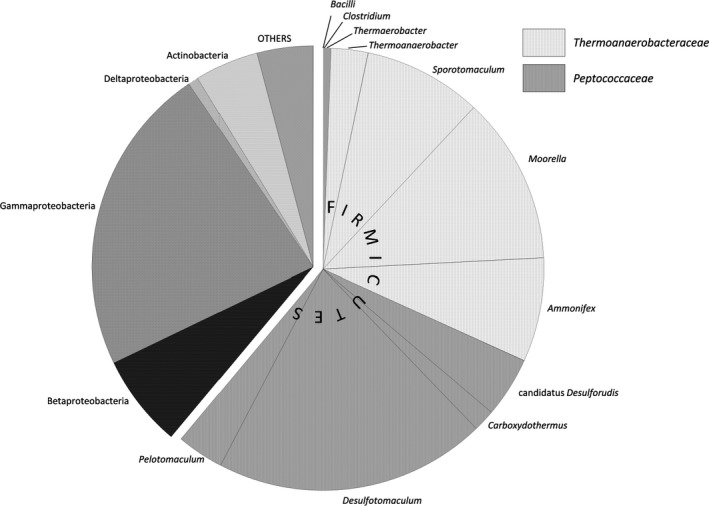
Composition of the bacterial community in the formation water collected from a deep aquifer (−853 m). The pie chart represents the percentage of each taxon within the whole microbial species analysed.

In the autumn of 2011, the FW of a deep aquifer (aquifer 1) used for geological storage of natural gas was sampled after cleaning of the sampling well, to remove the microorganisms growing as a biofilm on the tubing and the wellhead (see Basso *et al*., 2009 for Scheme of principle of a natural gas underground storage in an aquifer). During this study, the methane bubble was located close to the well and the presence of methane, originally dissolved at reservoir depth, was observed in the sampled water. At the time of sampling, the water was at a temperature of 36°C, with a pH of 8.25, and no oxygen was detected and a redox potential of −363 mV (Table 1). This aquifer displayed a total organic carbon concentration (TOC; 1 mg l^−1^) typical of this type of environment which has been little impacted by human activity (Pedersen, 1997; Sahl *et al*., 2008). Meckenstock *et al*. (2015) report in their review dedicated to microbial clean‐up in contaminated aquifers that only 0.5–5% of this TOC is likely to be directly used as carbon source by the microbial community which makes this deep aquifer an oligotrophic environment. The analysis of the chemical composition of this water reveals a sulphate content of 2186.5 mg l^−1^, a total iron content of 3200 μg l^−1^ but no nitrate. These data suggest that the indigenous microbial community is probably dominated by sulphate‐reducing bacteria. Sulphate reduction is indeed an important metabolic process in deep subsurface environments and particularly in aquifers (Detmers *et al*., 2004; Amend and Teske, 2005; Bombach *et al*., 2010; Itävaara *et al*., 2011) where it has been demonstrated that sulphate‐reducing bacteria also play a key role in the degradation of mono‐aromatic hydrocarbons (Basso *et al*., 2009; Berlendis *et al*., 2010; Aüllo *et al*., 2016). The concentrated biomass derived from the filtration of FW was subjected to a diversity analysis in order to determine the predominant bacterial groups. For this purpose, the V4 region of the sequence of the 16S rRNA gene was targeted by NGS (no archaea were detected). The taxa detected in this study are regularly found in studies of deep subsurface ecosystems. Figure 1 shows that Firmicutes were clearly predominant (61.1%) among the 62 OTUs found (similarity ≥ 97%), with 29.3% of *Peptococcaceae* (*Carboxydothermus*, candidatus *Desulforudis*,* Pelotomaculum* and *Desulfotomaculum*) and 31.2% of sequences belonging to *Thermoanaerobacteraceae* (*Thermoanaerobacter*,* Moorella*,* Sporotomaculum* and *Ammonifex*). The detection thermophilic microorganisms signatures (e.g. *Moorella* or *Thermoanaerobacter*) in a mesothermic environment may be surprising. However, similar results have already been reported in the study of another mesothermic aquifer (Berlendis *et al*., 2010; aquifer 2 in this study). Taking into account of the diversity observed, most sulphate reduction must be carried out by Firmicutes since the Deltaproteobacteria, which include a large number of sulphate‐reducing bacteria, represent less than 1% of total sequences. The dominance of the sulphate‐reducing community by Firmicutes has been shown in a 120 m deep aquifer displaying low sulphate concentrations (≤ 17.1 mg l^−1^; Detmers *et al*., 2004). Salinity seems to play a role in the distribution of Deltaproteobacteria and sulphate‐reducing Firmicutes (Leloup *et al*., 2005). Here, the dominance of Firmicutes can probably be further explained by their capacity to sporulate, their capacity to survive in oligotrophic environments and their metabolic versatility (Sass *et al*., 1997; Spring and Rosenzweig, 2006; Orsi *et al*., 2016). Moreover, some studies have suggested or demonstrated the key role of some *Peptococcaceae*, and in particular representatives of the genera *Desulfotomaculum*,* Desulfosporosinus* and *Pelotomaculum*, in the degradation of BTEX (Robertson *et al*., 2000; Liu *et al*., 2004; Morasch *et al*., 2004; Cupples, 2011; Abu Laban *et al*., 2015).

### Biodegradation assays

The day after sampling, assays of biodegradation of toluene and the 3 xylene isomers were initiated either directly on the water obtained from the site (FW+TX) or in water supplemented with concentrated indigenous bacterial biomass (FWCB+TX). A cell‐count carried out in FW using epifluorescence (DAPI) showed a cell concentration of 4.5 × 10^5^ ± 2.3 × 10^5^ cells ml^−1^, while the concentration of microorganisms in FWCB was four times greater; 1.8 × 10^6^ cells ml^−1^. While it is difficult to quantify microorganisms attached to the mineral matrix in deep aquifers, it is commonly accepted that the indigenous biomass growing in a biofilm is very largely dominant compared with pelagic microorganisms (Whitman *et al*., 1998; Griebler and Lueders, 2009). Even if the diversity between the pelagic and the attached microorganisms can differ (Röling and van Verseveld, 2002), the biomass supplemented condition tended to simulate the influence of the bacterial concentration in our biodegradation assays. In the two microcosms of this study, toluene and *m*‐ and *p*‐ xylenes (T*m*,* p*X) were degraded simultaneously (Fig. 1). Toluene is assumed to be the most easily biodegraded aromatic hydrocarbon in anoxic conditions, which induces that it is degraded first and therefore delay the degradation of other hydrocarbons (Haag *et al*., 1991; Edwards *et al*., 1992; Phelps and Young, 1999; Meckenstock *et al*., 2004; Morasch *et al*., 2004). However, this interpretation must be tempered since it is evident that the biodegradation potential depends on the pool of key genes present. Indeed simultaneous biodegradation of toluene and xylene has already been observed (Herrmann *et al*., 2009; Shah, 2014). It is interesting to observe that while the *m*‐ and *p*‐xylenes were degraded, *o*‐xylene resisted degradation throughout the incubation period. In the FW degradation assay, the degradation of T*m*,* p*X began after a lag phase of about 800 days. Degradation was almost four times faster (237 days) when the biomass was concentrated fourfold, which implies that the number of microorganisms influences T*m*,* p*X degradation. This result suggests that the in situ biodegradation process, with a supposed higher concentration of microorganisms in biofilm structures, could be faster than revealed by biodegradation studies in the laboratory (Botton and Parson, 2006; Berlendis *et al*., 2010; Higashioka *et al*., 2012; Aüllo *et al*., 2016). Indeed, only the pelagic fraction of the aquifers can be harvested, which underestimates the potential for in situ biodegradation.

### *BssA* detection assay by PCR

Numerous studies have shown that the *bssA* and *bssA*‐like genes can serve as biomarkers for the in situ anaerobic degradation of toluene and xylenes (Krieger *et al*., 1999; Andreoni and Gianfreda, 2007; Kazy *et al*., 2010; Cury *et al*., 2015). These studies are most frequently conducted in surface environments or in shallow aquifers. Even though the diversity study based on 16S rRNA gene indicates a predominance of Firmicutes and the water composition suggests that sulphate reduction could play a major role in this aquifer, the choice was made to be as exhaustive as possible regarding the metabolisms involved in bioattenuation. For this purpose, four primer pairs designed to target nitrate‐reducing bacteria (Brow *et al*., 2013), iron‐reducing bacteria (Staats *et al*., 2011), sulphate‐reducing bacteria belonging to the Proteobacteria or Clostridiales (Winderl *et al*., 2007; von Netzer *et al*., 2013) were tested as part of this study (Table S1). The *bssA* gene was not amplified by PCR in the DNA extracted from FW samples whether supplemented or not supplemented with concentrated biomass, although the degradation results obtained subsequently in the microcosms demonstrated the capacity to degrade the T*m*,* p*X. Our team has been trying to demonstrate the presence of this gene directly in FW for some years without success. Three times only this gene was amplified by the primers of Winderl *et al*. (2007) in microcosms from three different aquifers (aquifers 1, 2 and 3), after incubation periods of more than 100 days (data not shown). It should be noted that the amplicons corresponding to the expected size had to be systematically purified on an agarose gel in order to eliminate numerous non‐specific amplifications before sequencing. Although this amplification approach has proven its value in surface environments and shallow aquifers, it did not seem appropriate for the constraints of our study sites (trace mono‐aromatic hydrocarbons, low biomass and impossibility of recovering biofilms in this industrial context). Therefore, we had to develop a new molecular biological approach enabling detection of the *bssA* gene directly in FW while conducting time‐consuming biodegradation assays.

### *BssA* detection assay using sequence capture by hybridization approach

**Figure 2:**
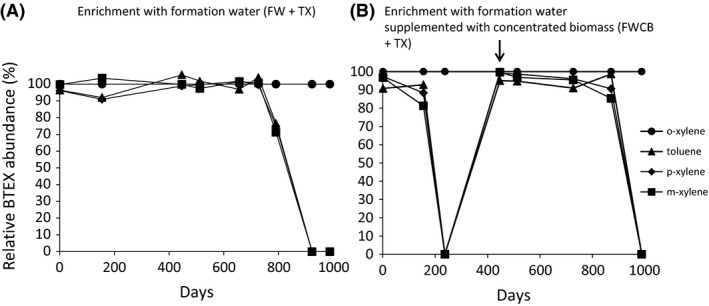
Degradation of mono‐aromatic hydrocarbons (toluene, *m*‐ and *p*‐xylenes) during incubation of formation water collected anoxically to protect autochthonous microbiota, FW (A) or with the formation water supplemented with concentrated biomass, FWCB (B). Filled circles: *o*‐xylene, filled triangles: toluene, filled diamonds: *p*‐xylene, filled squares: *m*‐xylene. Arrow indicates at day 447 the addition of toluene, *m*‐ and *p*‐xylenes (10 ppm). Start levels of mono‐aromatic hydrocarbons were 10 ppm.

In 2013, Denonfoux and co‐workers successfully combined a SHS method with next‐generation sequencing for the first time, in order to capture a biomarker gene in a complex metagenome. The methodology was used to explore the methanogenic communities present in a lacustrine environment by targeting the methyl coenzyme M reductase subunit A (*mcrA*) gene with a set of non‐overlapping probes, which targeted both known sequences and potential undescribed variants of the *mcrA* gene. The *mcrA* sequences represented more than 40% of the obtained sequences after two cycles of capture, revealing enrichment compared with shotgun sequencing, in which only 0.003% of the sequences corresponded to the target gene. In addition, because *mcrA* and 16S rRNA gene phylogenies are congruent, this approach allowed the methanogen community to be described and revealed higher diversity than previously observed with other methods. Indeed, hybridization capture recovered sequences from the Methanobacteriales order, belonging to the rare biosphere, which were not detected through direct sample sequencing due to the sequencing depth, or through PCR amplification, due to possible primer bias. This method appeared to be appropriate for our constraints given its sensitivity, and the fact that it does not need amplification step, which is often unfruitful in this type of study. The SHS probes (Table S1) were designed from *bssA* sequences of sulphate‐reducing bacteria belonging to the Deltaproteobacteria and the Firmicutes, as well as the three sequences previously amplified with the primers of Winderl *et al*. (2007) in enrichments with mono‐aromatic hydrocarbons (KX576575, KX576576, KX576577). After analysis of reads (Table S2), 498 500 reads proved close to *bssA* and *bssA*‐like genes and were grouped into three *bssA* homologous contigs. The contig sequence_10944 alone includes 98% of the *bssA* reads indicating that such *bssA* gene dominates in the ecosystem. Sequence capture by hybridization gives quantitative results close to that obtained by qPCR as demonstrates by Denonfoux *et al*. (2013). The three contigs obtained by sequence capture and the three *bssA* amplicons obtained in this study were compared with sequences from pure strains and environmental samples deposited in international databases after having been translated into AA sequences. The results are presented as a phylogenetic tree constructed from the comparison of a 90AA region. The main contig, contig sequence_10944 (aquifer 1) and the amplicons obtained from the sulphate‐reducing enrichments from FWs (aquifers 2 and 3) are closed to *bssA* sequences defined as sensu stricto by Acosta‐González *et al*. (2013). The dominant *bssA* gene obtained by SHS is close to the BF clone obtained from an enrichment described as degrading benzene and dominated by *Peptococcaceae*‐related Gram‐positive microorganisms (87% identity, 276AA). In our current state of knowledge, the reason for the presence of this gene in this enrichment described by Abu Laban *et al*. (2010) cannot be explained since the initial benzene biodegradation step does not seem to involve the addition of a fumarate molecule (enabled by the benzylsuccinate synthase) but a carboxylation as described by the authors. In our case, the procurement of a *bssA* gene associated with the *Peptococcaceae* family is consistent with the diversity data obtained in this study (Fig. 1) and tends to confirm the supposed role of *Peptococcaceae* in the degradation of T*m*,* p*X in this deep aquifer. In referring to the broad dominance of Firmicutes over Deltaproteobacteria, we can hypothesize that these are the principal sulphate‐reducing bacteria in this deep aquifer. Several studies have provided evidence for a positive correlation between the dominance of sulphate‐reducing bacteria affiliated to *Peptococcaceae* in subsurface environments and depth (Moser *et al*., 2005; Chivian *et al*., 2008; Itävaara *et al*., 2011; Guan *et al*., 2013). These microorganisms play a major role in the carbon cycle in deep environments via the recycling of organic material, which is, in our context, the degradation of mono‐aromatic hydrocarbons. To date, only two strains of *Peptococcaceae* (*Desulfotomaculum* sp. Ox39 and *Desulfosporosinus meridiei*) have been described as being able to degrade toluene and/or xylenes (Liu and Garcia‐Dominguez, 2004; Morasch *et al*., 2004). However, several studies using non culture‐based approaches tend to demonstrate that these microorganisms are often playing a key role in the degradation of mono‐aromatic hydrocarbons. In the case of a gas condensate‐contaminated aquifer, *Desulfosporosinus* sp. was shown to initiate toluene degradation (Fowler *et al*., 2012, 2014). The key role of the *Peptococcaceae* has also been demonstrated in other environments and in sulphate‐reducing conditions and/or methanogenesis (Abu Laban *et al*., 2009; Winderl *et al*., 2010; Pilloni *et al*., 2011; Sun and Cupples, 2012; Sun *et al*., 2014; Abu Laban *et al*., 2015; Tan *et al*., 2015). It is interesting to note that the *bssA* amplicon obtained for the enrichment culture with FW from the aquifer 1 at the end of degradation (Fig. 2) is phylogenetically located in the OX39‐homologues cluster as described by von Netzer *et al*. (2013). The genes present in this cluster could be involved in the degradation of xylenes (Herrmann *et al*., 2009; Bragalini *et al*., 2014). This sequence is close to the *Desulfotomaculum* sp. Ox39 *bssA*‐like gene (74% similarity, 227AA) and to environmental sequences obtained from enrichments derived from contaminated aquifers (Herrmann *et al*., 2009; von Netzer *et al*., 2013). Finally, the two last contigs that represent only 2% of the bssA homologous sequences obtained by SHS (Table S2), contig sequence_48572 and contig sequence_31410, form a separate cluster located between the *assA* and the *bssA* genes. The existence of deeply branching bacteria was also found in the DNA‐SIP study performed on a sample from the Testfeld Süd aquifer contaminated by hydrocarbons (Winderl *et al*., 2010), suggesting that a large part of the diversity of *bssA* sequences sensu stricto and sensu lato, and *bssA* homologues (*assA*,* nmsA*,* hbsA*) is still to be discovered. The contig sequence_48572 is close to the *Desulfobacula toluolica bssA* sequence (42% similarity, 115AA; ABM92935). As regard the contig sequence_31410 is close to a *Desulfotomaculum* sp. 46_20 alkylsuccinate synthase obtained from an oil reservoir in Alaska (46%/138AA; KUK63464), but also to a pyruvate‐formate lyase derived from a strain affiliated to *Peptococcaceae* from a Opalinus Clay rock porewater BRC‐3 borehole (39%/142AA; KJS47223) and to a glycyl radical enzyme of the strain *Desulfosporosinus* sp. BRH_c37, also obtained from the BRC‐3 site (38%/143AA; KUO70645).

**Figure 3:**
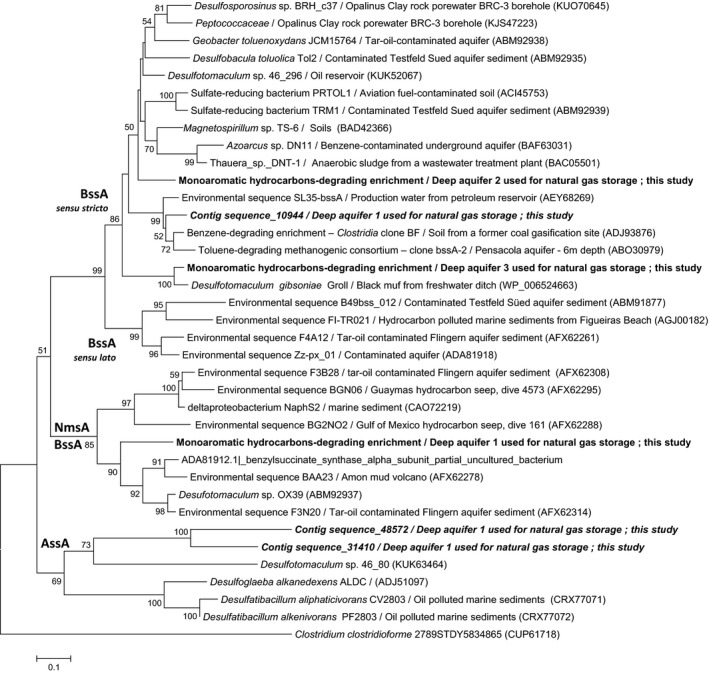
Phylogenetic tree based on partial *bssA*‐like amino acid sequences from deep aquifers used for geological natural gas storage (in bold; this study) compared with sequences from pure strains, enrichment cultures or environments retrieved in the databases. Sequences in bold were obtained by PCR from genomic DNA from mono‐aromatic hydrocarbon‐degrading enrichment cultures (aquifers 1, 2 and 3). Sequences in bold and italic were obtained by SHS method directly from formation water (aquifer 1). The evolutionary distances were computed using the Poisson correction method. Evolutionary analyses were conducted in MEGA6 with a bootstrap test of 1000 replicates.

## Conclusions

The study of the potential for TX degradation in oligotrophic environments such as deep aquifers is very difficult using conventional cultural approaches, as they require long incubation periods (several months to several years) and/or biomass. Biomarker detection allowing the evaluation of the biodegradation potential of an ecosystem or the monitoring of bioremediation operations is necessary tools for environmental engineering. Currently, the *bssA* and *bssA*‐like genes represent excellent biomarkers for the degradation of some mono‐aromatic hydrocarbons. However, no primer sets tested in the study enabled demonstration of the presence of *bssA* genes directly in FW, while enrichments subsequently showed that the metabolic potential was present.

Direct sequencing of metagenomic samples in recent years has allowed for increased precision in microbial diversity analyses but only dominant taxa could be revealed. The sequence capture by hybridization approach used in this study proved its efficiency for the specific capture of targeted *bssA* sequences. Indeed, this was the only method that enabled *bssA* and *bssA*‐like sequences to be obtained directly from FW. Therefore, this method constitutes a major asset in developing a clearer understanding of ecosystems and in monitoring bioattenuation phenomena in the context of mature environmental engineering. The *bssA* sequences and the diversity analyses based on the 16S rDNA sequences once again revealed the key role of *Peptococcaceae* in the degradation of mono‐aromatic hydrocarbons in deep continental aquifers.

## Conflict of interest

None declared.

## Supporting information


**Table S1.** Primers and gene capture probes used in this study.

**Table S2.** Summary statistics from *bssA* gene capture coupled to Illumina sequencing.
