# Supplementary material for: The sequence capture by hybridization: a new approach for revealing the potential of mono‐aromatic hydrocarbons bioattenuation in a deep oligotrophic aquifer
Source: Microb Biotechnol. 2016 Oct 21;10(2):469–79. doi: 10.1111/1751-7915.12426 (PMC5328808; doi:10.1111/1751-7915.12426)
Supplement: Supplementary file 1 — Table S1. Primers and gene capture probes used in this study. [file MBT2-10-469-s001.docx]

Table S1 : Primers and gene capture probes used in this study.

| **Primers set** | **Forward primer sequence** | **Reverse primer sequence** | **Reference** |
| --- | --- | --- | --- |
| 7768F/8543R | CAAYGATTTAACCRACGCCAT | TCGTCRTTGCCCCAYTTNGG | von Netzer *et al*., 2013 |
| 7772F/8546R | GACATGACCGACGCSATYCT | TCGTCGTCRTTGCCCCAYTT | Winderl *et al*., 2007 |
| bssA3F/bssAr | TCGAYGAYGGSTGCATGGA | GCTGCATTTCTTCGAAACCT | Staats *et al*., 2011 |
| 997F/1230R | CTGCTGTGGCCSTAYTACAAG | GATGGCGTCGGTCATGTCGKT | Brow *et al*., 2013 |
| **Probe** | **Sequence** | | **Reference** |
| S39 | CGARCAGWTGAAATATTAYRSCMAGTTCAGC | | **This study** |
| S410 | TCGTNTCCAGYATYGAYGACGGSTGCATGGAACTBGGC | | **This study** |
| S467 | CCGAGCAGRCSAAYGGBTGGCAYAACCCCATHACVACM | | **This study** |
| S529 | GCCATCAAGAARCTSATCTAYGAYGABAARAA | | **This study** |
| S1464 | TCSGAGCAGCCSAAYGGYTGGCAYAACCCCATYACCA | | **This study** |
