# Supplementary material for: The sequence capture by hybridization: a new approach for revealing the potential of mono‐aromatic hydrocarbons bioattenuation in a deep oligotrophic aquifer
Source: Microb Biotechnol. 2016 Oct 21;10(2):469–79. doi: 10.1111/1751-7915.12426 (PMC5328808; doi:10.1111/1751-7915.12426)
Supplement: Supplementary file 2 — Table S2. Summary statistics from bssA gene capture coupled to Illumina sequencing. [file MBT2-10-469-s002.docx]

**Supplementary Table S2 :** Summary statistics from bssA gene capture coupled to Illumina sequencing.

| Total number of pairs of reads | 6457686 | | |
| --- | --- | --- | --- |
| Number of reads after pre-processing | 6130281 | | |
| Average length of cleaned reads (bases) | 287 | | |
| bssA homologous sequences | 498500 | | |
| bssA homologous contigs | 3 | | |
| SHS Sequences used in this study | Contig | Length | Number of reads |
|  | c10944 | 831 | 489300 |
|  | s48572 | 350 | 1600 |
|  | s31410 | 467 | 7600 |
